# Sleep patterns among shift-working flight controllers of the International Space Station: an observational study on the JAXA Flight Control Team

**DOI:** 10.1186/s40101-016-0108-4

**Published:** 2016-09-01

**Authors:** Koh Mizuno, Akiko Matsumoto, Tatsuya Aiba, Takashi Abe, Hiroshi Ohshima, Masaya Takahashi, Yuichi Inoue

**Affiliations:** 1 Space Biomedical Research Group, Flight Crew Operations and Technology Unit, Human Spaceflight Technology Directorate, Japan Aerospace Exploration Agency, Tsukuba Space Center, 2-1-1 Sengen, Tsukuba, Ibaraki 305-8505 Japan; 2 Faculty of Education, Tohoku Fukushi University, 1-19-1 Kunimi, Aoba-Ku, Sendai, Miyagi 981-8523 Japan; 3 Research Center for Overwork-Related Disorders, National Institute of Occupational Safety and Health, 6-21-1 Nagao, Tama-Ku, Kawasaki 214-8585 Japan; 4 Department of Somnology, Tokyo Medical University, 6-7-1 Nishi-Shinjuku, Shinjuku-Ku, Tokyo 160-0023 Japan; 5 Japan Somnology Center, Institute of Neuropsychiatry, 91 Benten-Machi, Shinjuku-Ku, Tokyo 162-0851 Japan

**Keywords:** Space mission operation, Flight controller, Shift work sleep disorder, Actigraphy

## Abstract

**Background:**

Flight controllers of the International Space Station (ISS) are engaged in shift work to provide 24-h coverage to support ISS systems. The purpose of this study was to investigate the prevalence and associated factors of shift work sleep disorder (SWSD) among Japanese ISS flight controllers.

**Methods:**

A questionnaire study was conducted using the Standard Shiftwork Index to evaluate sleep-related problems and possible associated variables. Among 52 respondents out of 73 flight controllers, 30 subjects were identified as night shift workers who worked 3 or more night shifts per month. Those night shift workers who answered “almost always” to questions about experiencing insomnia or excessive sleepiness in any case of work shifts and days off were classified as having SWSD. Additionally, 7 night shift workers participated in supplemental wrist actigraphy data collection for 7 to 8 days including 3 to 4 days of consecutive night shifts.

**Results:**

Fourteen of 30 night shift workers were classified as having SWSD. Significant group differences were observed where the SWSD group felt that night shift work was harder and reported more frequent insomniac symptoms after a night shift. However, no other variables demonstrated remarkable differences between groups. Actigraphy results characterized 5 subjects reporting better perceived adaptation as having regular daytime sleep, for 6 to 9 h in total, between consecutive night shifts. On the other hand, 2 subjects reporting perceived maladaptation revealed different sleep patterns, with longer daytime sleep and large day-to-day variation in daytime sleep between consecutive night shifts, respectively.

**Conclusions:**

As the tasks for flight control require high levels of alertness and cognitive function, several characteristics, namely shift-working schedule (2 to 4 consecutive night shifts), very short break time (5 to 10 min/h) during work shifts, and cooperative work with onboard astronauts during the evening/night shift, accounted for increasing workloads especially in the case of night shifts, resulting in higher or equal prevalence of SWSD to that among other shift-working populations. Further studies are required to collect more actigraphy data and examine the possibility of interventions to improve SWSD.

## Background

The International Space Station (ISS) is a manned space facility where various scientific activities are conducted for scientific and technological advancement. By utilizing unique features of the space environment, a broad range of scientific experiments in fields including astronomy, materials science, life science, and space medicine have been performed in the ISS [1]. In addition, to ensure the first priority to maintain astronauts’ health and safety, astronauts’ onboard tasks include health-related activities such as physical exercise, as well as maintenance and repair of the complex ISS systems. Those activities are achieved by collaborative work between the onboard ISS astronauts and the flight controllers at mission control centers. Since problems or errors in space mission operation can produce huge economic losses or even loss of life, astronauts and flight controllers receive extensive basic and mission-specific training to acquire highly specific expertise for successful mission operation.

Among the issues in space mission operation, sleep loss has been considered a primary concern affecting onboard astronauts as well as flight controllers on the ground [2, 3]. Because both the astronauts and the flight controllers are engaged in tasks to monitor and operate complex equipment, decrements in alertness and cognitive function due to sleep loss might lead to inadequate performance and/or severe incidents. Furthermore, inadequate sleep is known to be associated with various adverse health consequences such as hypertension, obesity, digestive disorders, and depression. From these points of view, sleep studies for onboard astronauts have been conducted since the Skylab missions in 1970s, demonstrating shorter sleep duration and an increased incidence of disturbed sleep [2, 3]. However, research examining sleep patterns among flight controllers is very limited. To the best of our knowledge, only one preliminary study [4] has been conducted to examine sleep and performance among 17 flight controllers during a space shuttle mission, highlighting the need for more data to be collected on this issue, but no previous study has focused on flight controllers operating the ISS.

Sleep disturbances in onboard astronauts are thought to involve a combination of several factors including circadian misalignment due to relatively low light levels in the space vehicle, psychological excitation, space motion sickness, and temporal shifting in scheduled sleep time due to operational demands [3]. By contrast, as the ISS flight controllers are engaged in providing 24-h coverage to support ISS systems, shift work is the primary issue for sleep disturbance and decrements in alertness and cognitive function. Working night shifts is well known to associate with an increased risk of human error due to elevated sleepiness [5]. Moreover, the misalignment of working time with normal circadian phases often causes sleep deficiency with symptoms of insomnia, so-called shift work sleep disorder (SWSD) [6, 7]. Previous studies have demonstrated associations of SWSD with adverse effects such as an increased incidence of ulcers [8], symptoms of depression [8, 9], languidity [10, 11], and sleepiness-related accidents [8, 9].

Currently, in developed countries, shift work is a common system of work adopted for various types of jobs such as in medical, security, and transportation services; essential infrastructure maintenance; and industrial activities [12]. The behavioral characteristics of work differ across the types of shift work, ranging from physical (e.g., construction) to sedentary work (e.g., monitoring and/or operating technological devices). In addition, shift work schedules vary with regard to the duration of each shift, the direction of shift rotation (clockwise or counterclockwise), and the number of consecutive days of night shift work. Among various types of shift work, the shift work of the ISS flight controllers is primarily characterized by highly specific expertise to monitor and operate the complex equipment with high levels of vigilance and cognitive function. For performing well-coordinated work between the flight control team and onboard astronauts, communication ability and appropriate decision-making are also required.

In this study, we aimed to examine sleep patterns and conditions among the shift workers belonging to the Japan Aerospace Exploration Agency’s (JAXA) Flight Control Team (JFCT), along with their life habits and personality traits. In addition to the behavioral characteristics of work among ISS flight controllers, the shift work conducted by the JFCT includes three major considerations. First, because ISS operation is based on Greenwich Mean Time (GMT), Japan Standard Time (JST) for cooperative work between the JAXA mission control center and the ISS astronauts corresponds to the time from evening through midnight to early morning. Second, breaks during a work shift are limited to the time when telecommunication between the ISS and the JAXA mission control center is operationally interrupted, for 5 to 10 min/h. Third, in their rotating shift work schedule, the number of consecutive night shifts ranged from 2 to 4 days, which might increase the circadian stress compared to a rapid-rotation schedule. Thus, compared to other types of shift work, this combination of situations is both unique and challenging, especially during the night shift. We initially attempted to investigate the prevalence rate and associated factors of SWSD using a questionnaire. Subsequently, supplemental data collection using wrist actigraphy was conducted to obtain objective and detailed sleep information in order to explore the possible relation of SWSD with perceived adaptation to shift work.

## Methods

### Participants

Members of the JAXA Flight Control Team (JFCT) [13] who had been working at the mission control center to operate the Japanese experimental module (“Kibo”) on the ISS were the subjects of the present study. The mission control center is located at the Tsukuba Space Center in Ibaraki, Japan. As the primary role of Kibo among the ISS systems is to conduct various scientific experiments in the space environment, expertized 7 sections at the mission control center are responsible for around-the-clock operations to monitor and control the status of the experiments. The light intensity measured at eye level of the JFCT members at the mission control center was between 500 and 1000 lx.

During the period of the present study, there were 73 total registered members of the JFCT, with 7 to 13 members assigned to respective sections. The period for shifts under a 3-shift-per-day schedule was from 8 a.m. to 5 p.m. (day shift), from 4 p.m. to 1 a.m. (evening shift), and from midnight to 9 a.m. (night shift). The JFCT has been engaged not only in mission control but also in office work, training, and preparing for specific mission operations. The rotating shift schedule of the JFCT members was arranged differently in each of the 7 sections. One section adopted a schedule of 4 consecutive day shifts, evening shifts, and night shifts that were rotated at 8-day intervals. The schedule for the other 6 sections set 2 or 3 consecutive days for each work shift at relatively irregular intervals. Occasionally, the consecutive days of each work shift were decreased to 1 day or increased to 4 days due to vocational or personal reasons. Among those 6 sections, 1 section adopted a schedule where 2 or 3 consecutive days of night shifts were preceded by 1 to 3 consecutive days of evening shifts. The total number of night shifts among the JFCT members ranged from 0 to approximately 7 days/month.

### Questionnaire study

A questionnaire study was conducted using the Standard Shiftwork Index [14]. Although there has been no validation study of the Japanese version of the Standard Shiftwork Index, one previous study used the Standard Shiftwork Index to examine Japanese shift workers [15]. In this study, Japanese translation was carefully performed, and Cronbach’s alpha coefficients for the scales ranged between 0.67 and 0.91. We had a meeting with the JFCT members to explain the study and to deliver the questionnaire. For members who could not attend the meeting, the explanation was made individually. The members were instructed to answer regarding their status in the past 2 months, since identification of night shift workers was conducted by referring to the work attendance logs during the past 2 months. The questionnaire covered items including demographic variables, lifestyle habits, a subjective evaluation of work competency, sleep-related problems, and sleep activity patterns over a shift cycle. Questions regarding demographic items concerned age, sex, height, weight, length of work experience as a flight controller, one-way commute time, number of cohabiters, medical disorders under treatment, and medicines currently being taken. Lifestyle habits included smoking, drinking, consumption of caffeine, and exercise habits, as well as chronotype (morningness/eveningness). Smoker, habitual drinker, heavy caffeine drinker, and habitual exerciser were defined as smoking 1 or more cigarettes per day, drinking alcoholic beverages 4 or more days per week, drinking 5 or more cups of caffeinated beverages per day, and performing 30 min or more of physical exercise at least twice per week, respectively. Chronotype was evaluated using the diurnal scale [16], consisting of 7 items summarized for a total score. A higher score indicates a tendency toward morningness chronotype. Items for the subjective evaluation of work competency consisted of adaptation to shift work (1 = adapted, 2 = somewhat adapted, 3 = somewhat unadapted, 4 = unadapted), workload (1 = very easy, 2 = easy, 3 = moderate, 4 = hard, 5 = very hard), and risk of human error (1 = very low, 2 = low, 3 = moderate, 4 = high, 5 = very high). Questions about subjective workload and risk of human error were asked relative to day, evening, and night shifts. Sleep-related problems were assessed based on the following 7 items: difficulty falling asleep, difficulty maintaining sleep, waking up earlier than one’s intention, taking sleeping pills, using alcohol to help induce sleep, feeling unrestored after sleep, and excessive wake time sleepiness. For those items, the subjects were asked to respond with 1 of the 5 answer categories (1 = almost never, 2 = rarely, 3 = sometimes, 4 = frequently, 5 = almost always) for 4 conditions (during 3 work shifts and days off). Sleep activity patterns from 2 days before to 2 days after the shift work period were evaluated using 5- to 8-day logs. The logs were separately prepared in cases of day, evening, and night shifts to reflect typical patterns of each section’s shift schedule. The subjects were asked to describe the timing of sleep and naps on the log with a time resolution of 15 min. Consequently, bedtimes, rising times, and the length from bedtime to rising time in each of the 4 conditions (day, evening, and night work shifts and the night before a day off) could be evaluated. Those values in the case before a day off were read in the log for day shifts. In cases of night and evening shifts, as some subjects described taking naps between major sleep periods, the total time including major sleep and naps was calculated. In addition, as 2 or 3 h before habitual bedtime in the evening is characterized as having lowered sleep propensity (i.e., “sleep forbidden zone” [17]), subjects taking a nap before reporting to night shift duty (mostly between 7 p.m. and 11 p.m.) were identified as evening nappers.

The presence/absence of SWSD was judged based on criteria defined by the International Classification of Sleep Disorders (ICSD-2) [18]. According to the criterion used in previous studies [19, 20], night shift workers were defined as those who worked 3 or more night shifts per month. In the present study, we applied this criterion to identify night shift workers in the JFCT by referring to the work attendance logs during the past 2 months. If any of the responses among the night shift workers to insomnia-related items (difficulty falling asleep, difficulty maintaining sleep, waking up earlier than one’s intention, and feeling unrestored after waking from sleep) or excessive wake time sleepiness was scored as “almost always,” the respondent was categorized as SWSD−positive (SWSD+). Other night shift workers were identified as SWSD-negative (SWSD−). According to the work attendance logs, we identified two other groups: day workers (DW), who engaged in day shifts only, and occasional night and/or evening shift workers (ONEW), who had worked night shifts <3 days per month and/or evening shifts.

### Actigraphic study

Wrist actigraphy is an established procedure to investigate objective sleep-wake patterns using a watch-like device measuring wrist activity, which can record continuously for 24 h/day over long durations [21]. After the questionnaire study described above, an actigraphic study was conducted to observe objective information about sleep-wake patterns during the night shift period, which was considered to have significant relations with SWSD symptoms and perceived adaptation to shift work. In response to recruitment of JFCT night shift workers, 1 female and 6 male subjects (mean age 39.6 ± 7.2 years) participated in the study. These subjects also participated in the questionnaire study. The subjects were requested to wear an actigraph (Micro Motion Logger, Ambulatory Monitoring Inc., New York, USA) on the wrist of their non-dominant hand during the measurement period, except when bathing or performing vigorous physical exercise. Moreover, they kept a written sleep log to track their bedtime, waking time, naps, meal times, and times when the actigraph was temporarily removed. In this log, they also recorded any significant matters affecting their sleep. Additionally, in order to evaluate SWSD and their subjective work competency, the subjects answered the questionnaire similarly to the one used in the above questionnaire study.

The measurement period for actigraphy was planned from 2 days before the first night shift through 3 or 4 consecutive night shifts until the second day after the last night shift. As the shift schedule of 2 subjects included 1 or 2 days of consecutive evening shifts preceding 3 consecutive night shifts, the actigraphic recordings began 2 days before the first evening shift. Therefore, the planned measurement period ranged from 7 to 9 days for each respective subject. Within 2 weeks after the measurement, the subjects were individually debriefed on the actigraphic results with discussion of their actual circumstances.

Actigraphic recordings were analyzed with commercial software (Action-W, 2.4.20, Ambulatory Monitoring Inc., New York, USA) using the Cole-Kripke algorithm [22] for scoring sleeping/waking for every minute. Over the course of the recordings, time in bed (TIB, defined as the time in bed when subjects try to sleep) was determined according to the participants’ sleep logs. Subjects were asked to confirm TIB from the results of their own actigrams. After this confirmation, the software calculated the total time scored as sleep in TIB (TST, total sleep time) and the Sleep Efficiency Index (SEI, percentage of time spent sleeping per TIB). If the subjects took naps in addition to the major sleep period, the sum of TIB (STIB) and TST (STST) was calculated.

### Analysis

Statistical analysis was conducted using PASW Statistics software 17.0.2 (IBM Corporation) and “R” [23]. As multivariable analysis could not be applied to the questionnaire study results due to the small number of subjects, the effect of group was examined by one-factor ANOVA and Kruskal-Wallis test for continuous and ordinal variables, respectively. In the event that the group effect was significant, Bonferroni tests and Mann-Whitney’s *U* tests were used for pair-wise post hoc comparisons for continuous and ordinal variables, respectively. The Pearson chi-square test and Fisher’s exact test (in case the expected value in any group was less than 5) were used for categorical variables.

The actigraphic sleep parameters were analyzed by one-way repeated measures ANOVA to detect the effect of day-to-day variations. The level of significance was considered after the Greenhouse-Geisser correction for repeated measures. Bonferroni tests were used for pair-wise post hoc comparisons. Data on the initial night for 1 subject were missed due to unknown hardware trouble. In addition, the number of consecutive days of night shifts differed among the subjects (i.e., 3 days for 5 subjects, 4 days for 2 subjects). Therefore, statistical analysis was conducted for all 7 subjects for the day before the first night or evening shift, the days from the first to third night shifts, and the days after the last night shift. Statistical significance was defined as *p* < 0.05.

## Results

### Questionnaire study

Fifty-two (46 men and 6 women, mean age and SD 38.4 ± 6.2 years) of 73 total members of the JFCT completed the questionnaire, yielding a response rate of 71.2 %. Based on the records from work attendance logs for the past 2 months, 30 of 52 respondents were regarded as night shift workers who had worked at least 3 night shifts per month. The remaining 22 members consisted of 8 DW and 14 ONEW subjects. Among 30 night shift workers, 14 members (46.7 %) were identified as SWSD+, who answered “almost always” to any of the questions about experiencing symptoms related to insomnia or excessive wake time sleepiness. While DW subjects did not answer “almost always,” 7 ONEW members (50 %) answered “almost always” to any of those questions. Table 1 presents the demographic variables (age, sex, BMI, length of work experience as a flight controller, commute time, and conditions of cohabitating persons), lifestyle habits (chronotype, exercise, drinking, smoking, and consumption of caffeine), and average number of days working night or evening shifts per month according to subjects’ group assignment (DW, ONEW, SWSD−, and SWSD+). Among those variables, no significant effect of group was detected except on the existence of female subjects (*p* = 0.043) and the number of days of night and evening shifts per month (*p* < 0.0001 and *p* = 0.002, respectively). The DW group had a higher ratio of women (38 %) than the other groups. As the number of night shifts was used to determine subjects’ groups, significant differences were observed between night shift workers (SWSD+ and SWSD−) and the other two groups. Regarding the number of evening shifts, post hoc comparisons revealed no significant difference among the ONEW, SWSD−, and SWSD+ groups. The SWSD− and SWSD+ groups were similar in all measured variables, with no significant differences.

**Table 1:** Biographical variables, life habits, and number of night and evening shifts/month among the JFCT members

	DW (*n* = 8)	ONEW (*n* = 14)	Night shift worker		One-factor ANOVA or Fisher’s exact test	
			SWSD− (*n* = 16)	SWSD+ (*n* = 14)	*F* value	*p* value
Number of subjects who had symptoms of insomnia or excessive wake time sleepiness	0	7 (50 %)	0	14 (100 %)		
Age (years)	37.5 ± 7.6	39.8 ± 5.5	37.8 ± 5.8	38.1 ± 6.8	0.346	0.792
Number of female subjects	3	2	1	0		0.043
BMI	21.2 ± 2.2	22.1 ± 2.3	22.0 ± 1.6	21.8 ± 3.2	0.26	0.854
Length of experience as a flight controller (years)	2.2 ± 2.0	4.3 ± 0.9	3.8 ± 1.3	3.8 ± 1.2	1.886	0.145
One-way commuting time (min)	18 ± 12	23 ± 9	25 ± 18	28 ± 32	0.442	0.724
Number of subjects living alone	3 (38 %)	0	3 (19 %)	4 (29 %)		0.077
Number of subjects living with an infant	4 (50 %)	9 (64 %)	4 (25 %)	5 (36 %)		0.162
Diurnal score	16.0 ± 5.1	15.8 ± 2.9	16.3 ± 3.3	15.6 ± 2.3	0.098	0.961
Habitual exerciser	1 (13 %)	5 (36 %)	6 (40 %)	4 (29 %)		0.682
Habitual drinker	1 (13 %)	3 (21 %)	2 (13 %)	2 (14 %)		0.951
Smoker	0	0	1 (7 %)	2 (14 %)		0.473
Heavy caffeine drinker	1 (13 %)	2 (14 %)	4 (25 %)	1 (7 %)		0.716
Days of night shift/month	0	1.4 ± 0.9	4.9 ± 1.3	4.3 ± 1.4	49.44	<0.0001
Days of evening shift/month	0	4.0 ± 3.8	3.4 ± 1.5	3.6 ± 1.6	5.833	0.002

The values denote the mean ± standard deviation or the number of subjects and percentages. *p* values denote the results of the one-factor ANOVA or Fisher’s exact test among the subject groups

Table 2 shows the results of subjective work competency (adaptation to shift work, workload, and perceived risk of human error for each work shift) in each group. A significant group effect was observed for subjective workloads in the case of night shifts (*χ*^2^ = 7.53, *p* = 0.02). Post hoc comparisons revealed significantly higher workloads in the SWSD+ group than those in the SWSD− group. In addition, the SWSD+ group demonstrated tendencies to be subjectively unadapted and higher perceived risk of human error in the case of night shifts compared to the SWSD− group. There was no significant group difference in subjective workloads and perceived risk of human error for day and evening shifts.

**Table 2:** Comparison of subjective evaluation of work competency among the JFCT members

Adaptation to shift work							Kruskal-Wallis test	
		Adapted	Somewhat adapted	Somewhat unadapted	Unadapted		*χ* ^2^	*p* value
SWSD+		0.0 (0)	42.9 (6)	50.0 (7)	7.1 (1)		3.869	0.276
SWSD−		12.5 (2)	56.3 (9)	31.3 (5)	0.0 (0)			
ONEW		14.3 (2)	35.7 (5)	42.9 (6)	7.1 (1)			
DW		0.0 (0)	83.3 (5)	16.7 (1)	0.0 (0)			
Workload								
		Very easy	Easy	Moderate	Hard	Very hard		
Day shift	SWSD+	0.0 (0)	30.8 (4)	30.8 (4)	38.5 (5)	0.0 (0)	1.27	0.736
	SWSD−	0.0 (0)	6.3 (1)	68.8 (11)	25.0 (4)	0.0 (0)		
	ONEW	8.3 (1)	8.3 (1)	58.3 (7)	25.0 (3)	0.0 (0)		
	DW	16.7 (1)	16.7 (1)	50.0 (3)	16.7 (1)	0.0 (0)		
Evening shift	SWSD+	0.0 (0)	0.0 (0)	42.9 (6)	42.9 (6)	14.3 (2)	0.354	0.838
	SWSD−	0.0 (0)	6.3 (1)	31.3 (5)	62.5 (10)	0.0 (0)		
	ONEW	0.0 (0)	7.7 (1)	46.2 (6)	30.8 (4)	15.4 (2)		
Night shift	SWSD+^a^	0.0 (0)	0.0 (0)	7.1 (1)	28.6 (4)	64.3 (9)	7.533	0.027
	SWSD−	0.0 (0)	6.3 (1)	6.3 (1)	75.0 (12)	12.5 (2)		
	ONEW	9.1 (1)	0.0 (0)	9.1 (1)	54.5 (6)	27.3 (3)		
Perceived risk of human error								
		Very low	Low	Moderate	High	Very high		
Day shift	SWSD+	8.3 (1)	25.0 (3)	66.7(8)	0.0(0)	0.0 (0)	3.161	0.367
	SWSD−	0.0 (0)	25.0 (4)	62.5 (10)	12.5 (4)	0.0 (0)		
	ONEW	25.0 (3)	16.7 (2)	58.3 (7)	0.0 (0)	0.0 (0)		
	DW	0.0 (0)	16.7 (1)	83.3 (5)	0.0 (0)	0.0 (0)		
Evening shift	SWSD+	0.0 (0)	14.3 (2)	64.3 (9)	21.4 (3)	0.0 (0)	0.315	0.854
	SWSD−	0.0 (0)	12.5 (2)	56.3 (9)	31.3 (10)	0.0 (0)		
	ONEW	7.7 (1)	15.4 (2)	46.2 (6)	30.8 (4)	0.0 (0)		
Night shift	SWSD+	0.0 (0)	0.0 (0)	14.3 (2)	50.0 (7)	35.7 (5)	4.456	0.108
	SWSD−	0.0 (0)	0.0 (0)	31.3 (5)	62.5 (12)	6.3 (1)		
	ONEW	9.1 (1)	9.1 (1)	27.3 (3)	36.4 (4)	18.2 (2)		

The values denote the percentages of the subjects, with the raw number of subjects enclosed in *parentheses. p* values denote the results of the Kruskal-Wallis test

^a^Significantly different from SWSD− by post hoc comparison

**Fig. 1:**
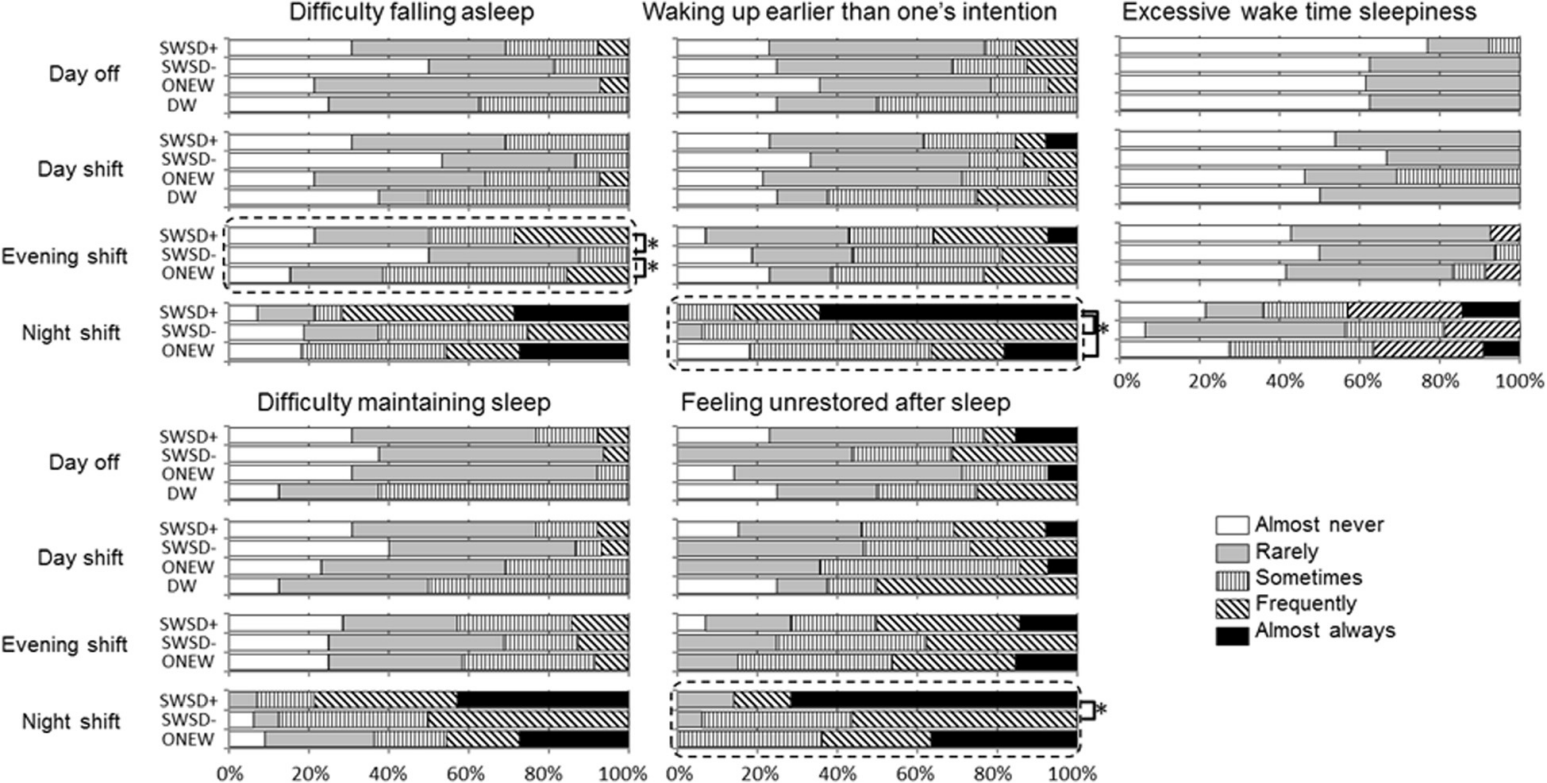
Comparison of insomnia symptoms and excessive sleepiness among the JFCT members. The percentages of responses are shown for each group. *Boxes* written by a *broken line* present a significant group effect detected by the Kruskal-Wallis test. *Significantly different by post hoc comparisons

Figure 1 presents the prevalence of symptoms of insomnia and excessive wake time sleepiness during the 3 work shifts and days off. For the 2 other items related to sleep-related problems, “taking sleeping pills” and “using alcohol to help induce sleep,” most of the respondents answered “almost never,” and no group difference was detected. Among the SWSD+ subjects, the most frequent insomnia symptoms to be answered “almost always” were “feeling unrestored after sleep” (10 subjects - 71 %,) and “waking up earlier than one’s intention” (9 subjects - 64 %) in the case of night shifts. Twelve of 14 SWSD+ subjects (86 %) answered “almost always” to items regarding insomnia symptoms only in the case of night shifts. Similar tendencies were observed in the ONEW subjects: the most frequent insomnia symptom to be answered “almost always” was “feeling unrestored after sleep” (4 of 11 subjects who had worked night shifts - 36 %), and the answer of “almost always” in response to items regarding insomnia symptoms was observed mostly in the case of night shifts (5 of 6 subjects [83 %] who had worked night shifts). The DW subjects did not answer “almost always” to those questions on the nights after day shifts and days off. Significant group differences were observed for the following three insomnia symptoms: “waking up earlier than one’s intention” (*χ*^2^ = 12.18, *p* = 0.002) and “feeling unrestored after sleep” (*χ*^2^ = 9.73, *p* = 0.008) in the case of night shifts and “difficulty falling asleep” (*χ*^2^ = 8.60, p = 0.014) in the case of evening shifts. Post hoc comparisons revealed that those symptoms were significantly more frequent in the SWSD+ group than in the SWSD− group. Furthermore, the ONEW group had a significantly higher reported frequency of “difficulty falling asleep” (*χ*^2^ = 8.60, *p* = 0.014) in the case of evening shifts compared to the SWSD− group and a significantly lower reported frequency of “waking up earlier than one’s intention” in the case of night shifts compared to the SWSD+ group.

Table 3 presents the sleep timing and duration in each work shift and day off. A significant group effect was found in the time of going to bed on nights before a day off [F (3, 43) = 4.268, p = 0.001]. The SWSD− group went to bed approximately 60 min later than the other groups, and post hoc comparison revealed a statistically significant difference from the ONEW group. This tendency for delayed sleep timing in the SWSD− group was demonstrated in the case of consecutive day shifts. There was no statistical difference in the rising times and sleep times across the groups. Regarding the parameters for evening and night shifts, there was no significant group difference among the ONEW, SWSD−, and SWSD+ groups, all of which included evening nappers in cases between consecutive night shifts (5 of 11 [45.5 %] ONEW subjects, 7 of 16 [43.8 %] SWSD− subjects, and 4 of 14 [28.6 %] SWSD+ subjects).

**Table 3:** Comparison of sleep timing and duration among the JFCT members

		DW	ONEW	SWSD−	SWSD+	One-factor ANOVA or chi-square test	
						*F* or *χ* ^2^ value	*p* value
Nights before days off	Bedtime (A)	23:28 ± 1:03	23:20 ± 1:03	0:28 ± 0:45 ^a^	23:28 ± 0:59	4.268	0.01
	Wake time (B)	7:15 ± 0:45	7:03 ± 1:09	7:40 ± 0:43	7:11 ± 0:51	1.257	0.301
	Length from A to B	7:47 ± 0:25	7:43 ± 0:50	7:12 ± 0:34	7:44 ± 1:04	1.623	0.198
Between consecutive day shifts	Bedtime (A)	22:56 ± 0:33	23:09 ± 0:46	23:43 ± 0:56	22:58 ± 0:56	2.54	0.068
	Wake time (B)	5:34 ± 0:40	6:09 ± 0:28	6:13 ± 0:36	5:53 ± 0:41	2.537	0.069
	Length from A to B	6:38 ± 0:21	7:00 ± 0:42	6:30 ± 0:54	6:55 ± 0:57	1.182	0.327
Between consecutive evening shifts	Bedtime (A)		3:25 ± 1:24	3:10 ± 0:51	3:21 ± 0:49	0.23	0.796
	Wake time (B)		9:30 ± 2:06	9:50 ± 1:44	9:28 ± 1:29	0.19	0.828
	Length from A to B		6:05 ± 1:07	6:39 ± 1:16	6:06 ± 0:59	1.233	0.302
	Total time allocated for sleep		6:18 ± 0:45	6:58 ± 0:58	6:28 ± 0:36	2.754	0.076
Between consecutive night shifts	Bedtime (A)		12:30 ± 1:54	12:17 ± 2:31	12:12 ± 2:25	0.054	0.948
	Wake time (B)		18:00 ± 2:40	17:36 ± 3:20	17:54 ± 3:52	0.054	0.947
	Length from A to B		5:30 ± 1:19	5:19 ± 1:40	5:42 ± 1:59	0.19	0.828
	Total time allocated for sleep		6:25 ± 1:25	6:42 ± 1:44	6:42 ± 1:15	0.152	0.860
	Percentages of evening nappers (napping after 7 p.m.)		45.5 % (5)	43.8 % (7)	28.6 % (4)	4.901	0.086

The values denote mean ± standard deviation or percentages and number of subjects. *p* values denote the results of the one-factor ANOVA or Pearson chi-square test among the subjects groups. The total time allocated for sleep is the sum of the major sleep period and additional naps

^a^Significantly different from ONEW

### Actigraphic study

According to responses to the questionnaire, 6 of the 7 subjects were identified as SWSD− because they did not answer “almost always” to items regarding insomnia symptoms and excessive wake time sleepiness. Among those 6 SWSD− subjects, 1 subject answered “somewhat unadapted” to the item asking about subjective adaptation to shift work, whereas the other 5 subjects answered “adapted” or “somewhat adapted.” One of the 7 subjects was identified as SWSD+ due to answering “almost always” to items asking about waking earlier than one’s intention and feeling unrestored after sleep in the case of night shifts. This SWSD+ subject answered “unadapted” to the question asking about subjective adaptation to shift work.

**Fig. 2:**
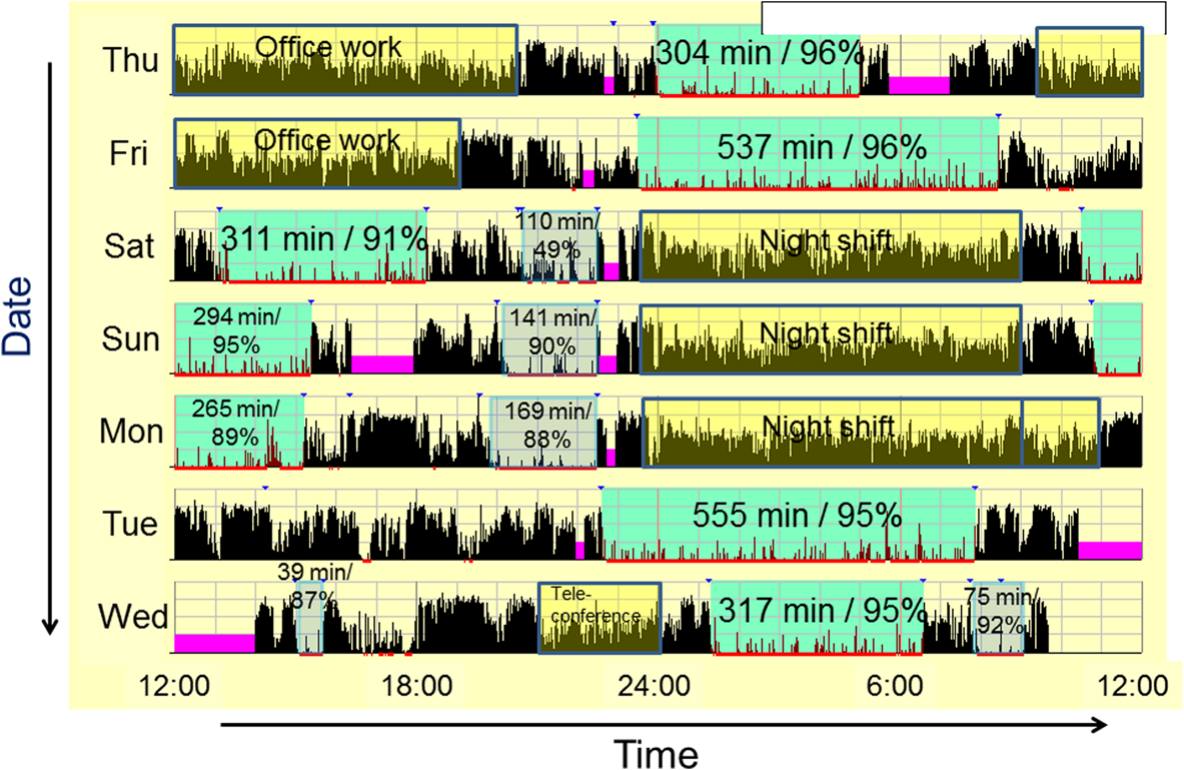
An example of actigraphic recording of a subject subjectively adapted to shift work. The *thin black vertical bars* denote activity counts per minute. *Colored areas* with numbers are described in minutes, and *percentages* denote the time when the subject was in bed attempting to fall asleep. Those numbers are TIB (time in bed when the subject was in bed) and SEI (Sleep Efficiency Index: percentage of time scored as sleep in TIB), respectively. *Red underlining* below each day’s horizontal axis denote the time scored as sleep. *Violet blocks* denote the time when the subject removed the actigraph. *Yellow blocks* denote the time when the subject was at work

**Fig. 3:**
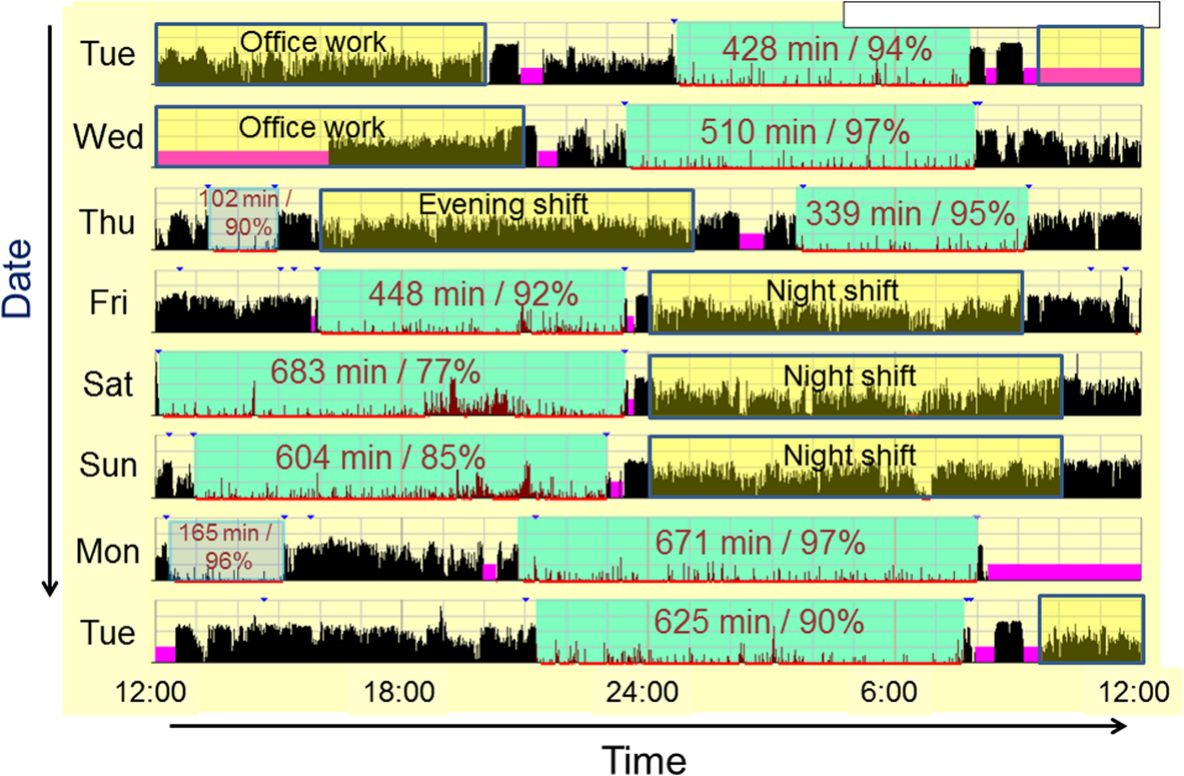
An example of actigraphic recording of a subject classified as having shift work sleep disorder. The meanings for the numbers and colors in the figure are as the same as those in Fig. 2

Figures 2 and 3 present two examples of actigraphic recordings. Figure 2 shows the example of a SWSD− subject subjectively self-evaluated as “somewhat adapted” to shift work. This subject slept twice (once in the daytime and once in the evening before reporting to night shift duty) between consecutive night shifts (on Sunday and Monday). This sleep pattern, involving taking naps before reporting to night shift duty, was observed in 3 of 7 subjects. The other 4 subjects, including 1 SWSD+ subject (Fig. 3) and 1 SWSD− subject who answered “somewhat unadapted” to subjective adaptation, showed a single major sleep period between consecutive night shifts. Figure 3 shows the SWSD+ subject, indicating long TIBs (>10 h) during and after a consecutive night shift period. Although relatively long awakenings appeared around 19:00 to 21:00 between consecutive night shifts (on Saturday and Sunday), it was confirmed that the subject was in bed trying to sleep, but could not sleep at those times. This subject revealed the lowest SEI (77 and 85 %) of TIB between consecutive night shifts, while the other subjects showed 87 to 98 % SEI in those daytime sleep periods. In cases of nocturnal sleep before and after the night shift period, all 7 subjects showed high SEI values ranging from 90 to 98 %.

**Fig. 4:**
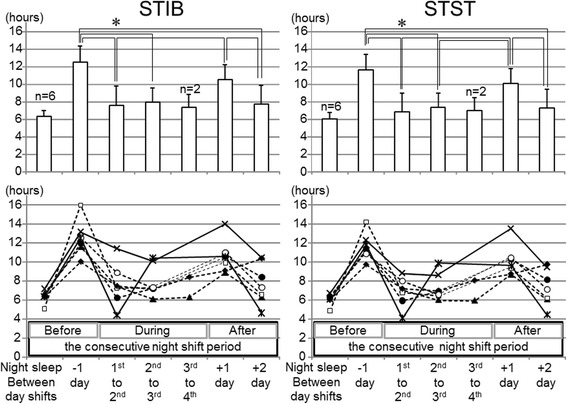
The sum of TIB (STIB) and the sum of TST (STST) before, during, and after a consecutive night shift period. The *upper* and *lower panels* show the mean ± SD and individual values, respectively. Post hoc comparison was performed on the days when the data from all subjects were obtained. *p < 0.05

Figure 4 presents STIB and STST before, during, and after night shifts. A significant effect of day-to-day variation was detected in both STIB [*F* (4, 24) = 12.50, *p* < 0.001] and STST [*F* (4, 24) = 14.23, *p* < 0.001]. As the subjects took daytime naps before reporting to the first night shift, STIB and STST were significantly increased on the day before going to the first night shift (*p* < 0.05). Significant increases in STIB and STST were also observed on the day after the last night shift because the subjects took daytime naps and/or had a long nocturnal sleep period (*p* < 0.05). Although those increases in STIB and STST on the days before and after the night shift period were consistently observed among all subjects, the values on the days between consecutive night shifts revealed individual characteristics suggesting possible association with subjective adaptation to shift work. Among the SWSD− subjects who answered “adapted” or “somewhat adapted” to shift work, consisting of 3 evening nappers and 2 non-nappers, STIB on the days between consecutive night shifts was relatively stable, ranging between 6 and 9 h (i.e., subjects indicated by dashed lines in Fig. 4). By contrast, the 2 subjects who answered “unadapted” and “somewhat unadapted” revealed different patterns of STIB between consecutive night shifts (i.e., subjects indicated by solid lines with cross and asterisk symbols in Fig. 4). One subject with long STIB is the SWSD+ subject shown in Fig. 3. The other subject showed large day-to-day variation in STIB; specifically, short (259 min) and long (621 min) STIB after the first and second night shifts, respectively.

## Discussion

The present study is the first to examine sleep among the shift workers who operate the Japanese experimental module, Kibo, on the ISS. Despite the significance of the work and difficult shift-working situations, only one study has examined sleep and performance among flight controllers, while operating a space shuttle mission [4]. However, as those flight controllers worked permanent day, evening, or night shifts through a 7-day space shuttle mission, the situation differed from that of the JFCT. A recent study reported sleep and cognitive functions of crewmembers and mission controllers during a ground-based simulation study for a 105-day space flight mission [24]. In that study, the mission controllers worked 24-h extended duration shifts and napped on 89 % of work shifts for an average duration of 3.4 h during the shift. Therefore, this situation was also not comparable to that of the JFCT.

To investigate detailed information about sleep-related problems, we administered the Standard Shiftwork Index [14] to inquire about those symptoms in each condition of work shifts and days off. Insomnia symptoms and decreased working competency in the SWSD+ group were observed mostly in the case of night shifts. In other words, subjective evaluations of insomnia symptoms and work competency revealed no group difference between day and evening shifts except difficulty falling asleep after evening shifts. It has been suggested that insomnia symptoms after night work could be a normal response [25], and that working night shifts is associated with an increased risk of human error due to elevated sleepiness [5]. Nevertheless, several characteristics of night shifts conducted by the JFCT might exacerbate insomnia symptoms and increase workloads. The number of consecutive night shifts adopted by the JFCT (i.e., generally 2 to 4 consecutive night shifts) is considered to be a major factor to increase the circadian stress associated with night shift work. As it was previously reported that daytime sleep after a night shift is typically shortened due to physiological and environmental reasons [5], consecutive night shifts over several days might accumulate a sleep debt with increasing fatigue and risk of human error [7, 25]. In the case of night shifts conducted by the JFCT, the tasks to monitor and operate the complex equipment might increase fatigue and risk of human error especially in the later night shifts of consecutive night shift periods. Such tendencies might be more severe in the SWSD+ subjects due to their more frequent insomnia symptoms compared to SWSD− subjects. Indeed, previous studies have demonstrated significant associations between SWSD and experience of accidents/errors [8, 9]. Notably, 5 of 11 subjects who had worked night shifts in the ONEW group answered “almost always” to items regarding insomnia symptoms in the case of night shifts. Although their night shifts numbered less than 3 days/month, fatigue and risk of human error in the later night shifts during periods of consecutive night shifts might be equal to those in the SWSD+ group.

The other factor that could increase the subjective workload for night shift workers was the cooperative work with ISS astronauts from evening to early morning. As this situation is due to the 9-h time difference between the JAXA mission control center (JST) and the ISS (GMT), the work for the JFCT during evening and night shifts includes direct communication with the onboard astronauts for performing real-time operations including the experimental implementation and troubleshooting of problems or anomalies. By contrast, JAXA day shifts (from 8 a.m. to 5 p.m. JST) correspond to the time for astronauts’ nocturnal sleep and post-sleep activities (from 10 p.m. to 7:30 a.m. GMT, i.e., from 7 a.m. to 4:30 p.m. JST). These differences in work characteristics between the day and evening/night shift could account for increased subjective workloads during evening/night shifts. It is conceivable that the SWSD+ subjects with sleep deficiency during the night shift periods felt the impact of night shifts more profoundly than the SWSD− subjects.

The DW group, consisting of 8 subjects who worked day shifts only, did not answer “almost always” to any of the items concerning insomnia symptoms or excessive wake time sleepiness. Previous studies investigating general populations have demonstrated that 10 % [11] and 18 % [8] of day workers reported symptoms of insomnia or excessive sleepiness. Taking this information into account, Drake et al. [8] estimated the differential prevalence (i.e., “true prevalence”) of SWSD in shift workers by subtracting that of day workers and reported that the raw and true prevalence of SWSD in shift workers were 28 and 10 %, respectively. From this point of view, although the number of DW subjects was small, it might suggest that the day shift for flight control at the mission control center did not cause insomnia or excessive wake time sleepiness. As we identified 47 % (14 of 30 subjects) of the JFCT night shift workers as SWSD+, this might represent a “true prevalence,” and symptoms of insomnia and higher subjective workloads were associated with night shifts due to the additional factors of the JFCT shift-working situation.

The present study identified SWSD+ subjects based on the criteria of answering “almost always” to any of the questions concerning insomnia or excessive wake time sleepiness. While no validated instrument is currently available to screen for SWSD, several major studies investigating the prevalence of SWSD in nurses [9, 10] and the general population [11] adopted criteria for SWSD as respondents answering “yes” to 3 questions about (1) the presence of either insomnia or excessive sleepiness, (2) the relation of those sleep problems to a night work schedule, and (3) the persistence of those sleep problems for at least 1 month. Those studies demonstrated that the prevalence of SWSD was 44.3 % [10] and 20.3 % [9] among nurses working 3 shifts, 28.9 % [10] and 24.4 % [9] for nurses working 2 shifts, and 32.1 % among rotating shift workers (working an unknown number of shifts) [11]. Another study [19] investigating police officers used different questionnaires and different criteria for identifying SWSD among those experiencing both excessive sleepiness and insomnia. As a result, 14.5 % of police officers who worked night shifts met the criteria. Interestingly, when the ICSD-2 criteria (experiencing insomnia or excessive sleepiness) were applied, the percentage increased dramatically to 54 %. Overall, although different criteria for identifying SWSD have been used among the studies, the prevalence of SWSD among JFCT night shift workers might be higher than or at least equal to those reported among other shift-working populations. If we categorized the subjects who answered “frequently” as SWSD+, the prevalence of SWSD+ increased to 87 % (26 of 30 subjects).

A previous study [10] investigating a relatively large number of nurses (*n* = 1968) found associations between SWSD and older age, male sex, and number of nights worked using logistic regression analysis. Comparison between the SWSD+ and SWSD− groups in the present study revealed no difference in those variables, suggesting that those factors might not be associated with SWSD in this specific population. Among demographic and lifestyle variables, the only variable associated with SWSD was the later bedtime of SWSD− subjects in the cases of day shifts and days off, suggesting that SWSD− subjects might tend to favor late hours. However, this speculation is inconsistent with the diurnal score results, which showed no difference in chronotype among the groups. As diurnal score was not a factor associated with SWSD in a study that examined nurses [10], chronotype might not be associated with SWSD among the JFCT members.

As we could not identify definitive associated factors of SWSD in the questionnaire study, an actigraphic study was additionally conducted to obtain objective, real-world information on sleep patterns to examine possible associations with SWSD or subjective adaptation to shift work. Because of the busy and important vocational duties in the JFCT under a nonstandardized shift schedule, this study was limited by issues including the small number of subjects, different night shift schedules among the subjects, lack of objective measurements evaluating cognitive functions, and possible factors under real-life conditions that might affect the results. However, the results may suggest characteristics in sleep patterns for coping with consecutive night shifts. As shown in Fig. 4, STIB on the days between consecutive night shifts demonstrated different characteristics in relation to subjective adaptation to the shift work. On those days, 5 SWSD− subjects with better subjective adaptation to shift work (indicated by a dashed line) showed relatively stable STIBs of 6 to 9 h with high SEI (87 to 98 %). These results suggest that one important strategy for coping with consecutive night shifts may be to make time window(s) for sleep as long as one’s ordinary night sleep. Notably, 3 of those 5 subjects were evening nappers who slept twice (once in the daytime and once in the evening before reporting to night shift duty) between consecutive night shifts. As in the questionnaire study, SWSD− subjects included both evening nappers and non-nappers. It seems that sleep patterns on the days of a night shift period might vary according to individual circumstances, including physiological characteristics related to sleep propensity, home life, and work situations. The subjects who adapted to shift work were considered to empirically learn effective strategies for maintaining sufficient sleep time on the days of a night shift period. As has been suggested [26], the appropriate strategy might depend on genetic characteristics involved in sleep-wake, circadian, and cognitive regulation.

It is conceivable that the measured sleep patterns of the remaining 2 subjects between consecutive night shifts were related to their subjective evaluations of SWSD+ and/or lower adaptation. The long daytime TIB with relatively long period of wakefulness (i.e., low SEI) in the SWSD+ subject might induce feelings of waking earlier than one’s intention and/or less refreshment after sleep. Short TIB after the first night shift in the other subject might bring about severe sleepiness and fatigue during the second night shift. As the debriefing confirmed that short TIB after the first night shift occurred because this subject worked on other tasks, such experiences could be likely to induce a feeling of maladaptation to shift work. Because 14 of 30 JFCT night shift workers were identified as SWSD+, there might be other sleep patterns specific to SWSD or low subjective adaptation to shift work. Therefore, further actigraphy data collection and examination of the possibility of interventions to improve SWSD is required in future studies.

Considering possible interventions for the JFCT, current working conditions involve several operational constraints. It has been suggested that rapid shift rotation is preferable to reduce the circadian stress associated with night shift work [27], but such scheduling was not adopted by the JFCT due to constraints imposed by their work other than flight control and for better working efficacy in order to operate and support experimental space missions within the same period over several consecutive days. An environmental issue for the JFCT is light intensity in the mission control center. Bright light is a strong factor for increasing one’s state of alertness [28], and the measured light intensity in the mission control center (between 500 and 1000 lx) is in the range where the alerting effect of light sharply decreases [28]. Although increasing environmental light intensity might be a countermeasure to overcome sleepiness during night shifts, it is not a suitable option because it disturbs the visibility of large displays on the walls of the mission control center. Another issue is the very short break time (5 to 10 min/h) during work shifts when the flight controllers must remain vigilant in their monitoring and operation duties. Although a study [29] on air traffic controllers reported that a 40-min scheduled naptime during night shifts is effective in improving psychomotor performance and alertness, the current circumstances of the JFCT do not allow such a time window for napping. As it is unclear if flight controllers working in mission control centers in other countries are confronted with similar issues regarding shift work, to establish an appropriate shift work system including development of possible countermeasures is essential for future space missions, and might be helpful for the other analogous types of shift work such as that spanning coordination across multiple time zones or involving highly sophisticated technical monitoring.

## Conclusions

In conclusion, the present study demonstrated the possibility of a higher or equal prevalence of SWSD among JFCT shift workers compared to other populations engaged in shift work. As insomnia symptoms and declining working competency were observed especially in the case of night shifts, a combination of several factors including the number of consecutive night shifts and working conditions are considered to make night work more challenging. While the questionnaire results indicated almost no difference in demographic and lifestyle variables between SWSD+ and SWSD− subjects, the results of actigraphic recordings indicated that subjects who were subjectively adapted to shift work maintained more regular and appropriate daytime sleep durations between consecutive night shifts. Those sleep patterns seemed to vary according to individual circumstances, which may reflect genetic characteristics and social situations. Given the small number of subjects who participated in the actigraphic study, a larger sample size is required to identify broader trends in sleep patterns.

The present results represent the first reference data on sleep patterns and conditions of the ISS flight controllers. As ISS mission control is operated internationally by mission control centers located in the USA, Russia, Germany, and Japan, investigating and comparing sleep conditions in shift-working situations across these mission control centers could contribute to establishing an appropriate shift work system according to the circumstances of each country.

## Abbreviations

DW: day workers
ICSD-2: International Classification of Sleep Disorders 2nd edition
ISS: International Space Station
JAXA: Japan Aerospace Exploration Agency
JFCT: JAXA Flight Control Team
ONEW: occasional night and/or evening shift workers
SEI: Sleep Efficiency Index
STIB: sum of TIB
STST: sum of TST
SWSD: shift work sleep disorder
SWSD−: SWSD-negative
SWSD+: SWSD-positive
TIB: time in bed
TST: total sleep time
